# Supplementary material for: Analysis of PPARγ Signaling Activity in Psoriasis
Source: Int J Mol Sci. 2021 Aug 10;22(16):8603. doi: 10.3390/ijms22168603 (PMC8395241; doi:10.3390/ijms22168603)
Supplement: Supplementary file 1 [file ijms-22-08603-s001.zip › Supplemental materials_Analysis of PPARg signaling activity in psoriasis/Pathway models/Models images and html files/Interleukin-17 and interleukin-22 signaling in psoriasis/11614.html]

TRAF6


# Protein TRAF6

|  |  |
| --- | --- |
| URN | urn:agi-llid:7189 |
| Total Entities | 0 |
| Connectivity | 2646 |
| Name | TRAF6 |
| Description | TNF receptor-associated factor 6, E3 ubiquitin protein ligase |
| Notes | The protein encoded by this gene is a member of the TNF receptor associated factor (TRAF) protein family. TRAF proteins are associated with, and mediate signal transduction from, members of the TNF receptor superfamily. This protein mediates signaling from members of the TNF receptor superfamily as well as the Toll/IL-1 family. Signals from receptors such as CD40, TNFSF11/RANCE and IL-1 have been shown to be mediated by this protein. This protein also interacts with various protein kinases including IRAK1/IRAK, SRC and PKCzeta, which provides a link between distinct signaling pathways. This protein functions as a signal transducer in the NF-kappaB pathway that activates IkappaB kinase (IKK) in response to proinflammatory cytokines. The interaction of this protein with UBE2N/UBC13, and UBE2V1/UEV1A, which are ubiquitin conjugating enzymes catalyzing the formation of polyubiquitin chains, has been found to be required for IKK activation by this protein. This protein also interacts with the transforming growth factor (TGF) beta receptor complex and is required for Smad-independent activation of the JNK and p38 kinases. This protein has an amino terminal RING domain which is followed by four zinc-finger motifs, a central coiled-coil region and a highly conserved carboxyl terminal domain, known as the TRAF-C domain. Two alternatively spliced transcript variants, encoding an identical protein, have been reported. [provided by RefSeq, Feb 2012] |
| Primary Cell Localization | Cytoplasm |
| ObjectType | Protein |

---

|  |  |
| --- | --- |
| Pathway | Osteoarthritis |
|  | Fatty Acids Block TLR Signaling in Enteroendocrine Cell |
|  | I-cell: CCK-8 Secretion and Regulation of Eating Behavior |
|  | Toll-like Receptors Act through MYD88-TIRAP Signaling |
|  | Antiviral Signaling through Pattern Recognition Receptors |
|  | Toll-like Receptors Act through MYD88 Signaling |
|  | TLR4 Signaling in Leukocytes |
|  | T-Cell Dependent B-Cell Activation |
|  | T-Cell Independent B-Cell Activation |
|  | V(D)J Recombination Activation |
|  | Immunoglobulin Class-Switch Recombination Activation |
|  | T-Cell Maturation (Hypothesis) |
|  | CD8+ T-Cell Activation |
|  | Th1-Cell Differentiation |
|  | Treg-Cell Differentiation |
|  | Th2-Cell Differentiation |
|  | T-Cell Receptor Signaling |
|  | Th17-Cell Differentiation |
|  | Toll-like Receptors in Sterile Inflammation |
|  | Toll-like Receptor Independent Sterile Inflammation |
|  | MC1R in anti-Inflammatory Signaling |
|  | Prostaglandin E2 Receptor Signaling in Neurons |
|  | Basophil Activation |
|  | Eosinophil Survival by Cytokine Signaling |
|  | Mast-Cell Activation without Degranulation through TNFSF8 Signaling |
|  | Mast-Cell Activation via IgE Signaling |
|  | Mast-Cell Activation without Degranulation |
|  | Mast-Cell Activation without Degranulation through IL33/IL1RL1 Signaling |
|  | Mast-Cell Activation without Degranulation through IL1R1 and TLR Signaling |
|  | Macrophage M1 Lineage |
|  | CD16/CD14 Proinflammatory Monocyte Activation |
|  | AHR Signaling in M1 Macrophages Function |
|  | anti-Inflammatory Function of Macrophage M2 Lineage |
|  | AXL Receptor Inhibits Macrophages and Dendritic Cells Function |
|  | Neutrophil Recruitment and Priming |
|  | Negative Acute Phase Proteins Synthesis |
|  | Positive Acute Phase Proteins Synthesis |
|  | Vascular Endothelial Cell Activation by Cytokines |
|  | Vascular Endothelial Cell Activation by Blood Coagulation Factors |
|  | Adipokines Production by Adipocyte |
|  | GFs/TNF -> Ion Channels |
|  | IL1B Expression Targets -> Nociception |
|  | Circadian Clock Genes in Suprachiasmatic Nuclei Neurons |
|  | Proteins Involved in Atherosclerosis |
|  | Scavenger Receptor OLR1 in Inflammation Related Endothelial Dysfunction |
|  | Dendritic Cells Function in Atherosclerosis |
|  | RAGE/AGER and S100 Proteins in Cardiovascular Injury |
|  | Th1-Cells Activation and Proliferation in Atherosclerosis |
|  | Myocardial Infarction |
|  | MAVS in Antiviral Innate Immune Response in Myocarditis |
|  | Lymphocyte Mediated Myocardial Injury in Myocarditis |
|  | Toll-like Receptors (TLR) in Antiviral Innate Immune Response |
|  | Inflammatory Reaction in Acne Vulgaris |
|  | Keratinocyte Proliferation in Acne Vulgaris |
|  | Atopic Dermatitis Onset |
|  | Mast-Cells Activation in Atopic Dermatitis |
|  | IL17 Signaling in Psoriasis |
|  | Dendritic Cells Function in Psoriasis |
|  | Apoptotic Keratinocytes Clearance Recession in Systemic Lupus Erythematosus |
|  | Dendritic Cell Activation in Systemic Lupus Erythematosis |
|  | Th17-Cell Function in Systemic Lupus Erythematosus |
|  | B-Cell Activation in Systemic Lupus Erythematosus |
|  | Th2-Cell Function in Systemic Lupus Erythematosus |
|  | Neutrophil and Macrophage Function in Systemic Lupus Erythematosus |
|  | Acute Cytotoxic CD8+T-Cell Response against Melanocytes |
|  | Vitiligo |
|  | T-Cell Cytotoxic Response against Melanocytes in Vitiligo |
|  | Humoral Immunity in Vitiligo |
|  | Treg-Cell Function in Diabetes Mellitus Type 1 |
|  | Natural Killer T-Cell Roles in Diabetes Mellitus Type 1 |
|  | non-Suppressive Treg-Cell in Diabetes Mellitus Type 1 |
|  | Plasmacytoid Dendritic Cell in Diabetes Mellitus Type 1 |
|  | TLR2 Signaling in Treg-Cell in Type 1 Diabetes (Animal Model) |
|  | Toll-like Receptors in beta-Cell |
|  | Peripheral Tolerance to Autoantigens Recession in Diabetes Mellitus Type 1 |
|  | TLR7 and TLR9 in Plasmacytoid Dendritic Cell |
|  | Conventional Dendritic Cell Role in Diabetes Mellitus Type 1 |
|  | Treg-Cell Activation in Diabetes Mellitus |
|  | Insulin Resistance in Myocytes Induced by Obesity |
|  | Hepatic Stellate Cells in non-Alcoholic Fatty Liver Disease |
|  | beta-Cell Destruction through Cytokines in Diabetes Mellitus |
|  | beta-Cell Destruction in Diabetes Mellitus Type 1 |
|  | Bone Remodeling in Hyperthyroidism |
|  | Adipokines Production by Adipocyte Impaired in Obesity |
|  | CD4+T-Cell Response in Celiac Disease |
|  | Celiac Disease |
|  | Crohn's Disease |
|  | Dendritic Cell Dysfunction in Crohn's disease |
|  | Dendritic Cell Function in Ulcerative Colitis |
|  | Epithelial Cell in the Innate Immune Response in Ulcerative Colitis |
|  | Nicotine anti-Inflammatory Effect in Ulcerative Colitis |
|  | Th2-Cell Function in Ulcerative Colitis |
|  | T-Cell Cytotoxic Response in Ulcerative Colitis |
|  | Antiphospholipid Antibodies in Endothelial Cell |
|  | B-Cell Chronic Lymphocytic Leukemia |
|  | Diffuse Large-B-Cell Lymphoma |
|  | Diffuse Large-B-Cell Lymphoma ABC Subtype |
|  | Follicular Lymphoma |
|  | Hodgkin Lymphoma |
|  | Mantle Cell Lymphoma |
|  | mTOR/NF-kB/BCR Signaling Disregulation in Mantle Cell Lymphoma |
|  | Mucosa-Associated Lymphoid Tissue (MALT) Lymphoma |
|  | Splenic Marginal Zone Lymphoma |
|  | TNFR/NF-kB Classical Pathway in Multiple Myeloma Plasma Cells |
|  | Osteoclast Activation in Multiple Myeloma |
|  | Multiple Myeloma |
|  | Proteins Involved in Multiple Myeloma |
|  | Antiphospholipid Antibodies and Endothelial Cell Interaction |
|  | T-Cell Acute Lymphoblastic Leukemia |
|  | Eosinophil Survival in Asthma |
|  | Il17 Signaling Related Neutrophilia in Asthma |
|  | IL33 Signaling Related Eosinophilia in Asthma |
|  | TSLP Signaling in Bronchial Epithelial Cell in Asthma |
|  | IL17F Signaling in Bronchial Epithelial Cell in Asthma |
|  | Club-Cell Role in Asthma |
|  | Basophil Activation in Asthma |
|  | Antigen-Presenting Cell Role in Asthma |
|  | B-Cell Mediated IgE Production in Asthma |
|  | Th17-Cell Differentiation in Asthma |
|  | Th2-Cell Response in Asthma |
|  | Mucin Production in Goblet Airway Epithelial Cells |
|  | CFTR Facilitates Oxidative Stress in Airway Epithelium in CF |
|  | Airway Epithelial Cell Dysfunction |
|  | CFTR Expression in Epithelial Cells (Class I Mutations) |
|  | Osteoclast Activation in Gout |
|  | Neutrophil Recruitment in Sinovium in Gout |
|  | Monocytes Function in Gout |
|  | Hyper IgM Syndrome |
|  | TNF and IL1B Induce Metalloproteinase Synthesis in Osteoarthritis |
|  | IL1B Induced Arthralgia |
|  | Dendritic Cell Dysfunction in Psoriatic Arthritis |
|  | Th17-Cell and Th1 Immune Responsein Psoriatic Arthritis |
|  | Osteoclast Activation in Psoriatic Arthritis |
|  | Synovial Fibroblast Activation in Psoriatic Arthritis |
|  | Keratinocyte Activation in Psoriatic Arthritis |
|  | PTPN22 Role in Rheumatoid Arthritis |
|  | Osteoclast Activation in Rheumatoid Arthritis |
|  | Synovial Fibroblast Activation by Citokines in Rheumatoid Arthritis |
|  | TLR2 Induced Synovial Fibroblast Activation in Rheumatoid Arthritis |
|  | Autocrine Cytokine/Chemokine Loops in Systemic Scleroderma |
|  | B-Cells Function in Systemic Scleroderma |
|  | Th2-Cells Function in Systemic Scleroderma |
|  | Dentin Formation Disruption |
|  | Th17-Cell Numbers Reduction in HIV |
|  | CD4+ T-Cell Function Decline in HIV |
|  | CD4+ T-Cell Death in HIV |
|  | Toll-like Receptors (TLR) in Periodontitis |
|  | IL1 Receptor Antagonist (IL1RN) Deficiency (DIRA) |
|  | PAPA Syndrome |
|  | TNF Receptor Associated Periodic Syndrome |
|  | Mevalonate Kinase Deficiency |
|  | Blau Syndrome |
|  | IL36 Receptor Antagonist (IL1F5) Deficiency (DITRA) |
|  | Cryopyrin (NLPR3) Associated Periodic Syndromes |
|  | Familial Cold Autoinflammatory Syndrome 2 |
|  | Familial Mediterranean Fever |
|  | Majeed Syndrome |
|  | Gluten Impact on Neuronal System (Hypothesis) |
|  | Osteoclasts Function in Osteopetrosis |
|  | Proteins Involved in Osteopetrosis |
|  | Osteoclast Activation in Postmenopause |
|  | Microglia Activation in Alzheimer's Disease |
|  | HMGB1 and IL1B in Neuroinflammation |
|  | Growth Factor Signaling in Neuroblastoma |
|  | Neuroblastoma |
|  | Microglia Activation in Parkinson's Disease |
|  | Subepithelial Fibroblasts in Middle Ear Cholesteatoma |
|  | Cholesteatoma |
|  | HPV E6 and E7 Induced Disruption of TNFR/NF-kB Signaling in Keratinocytes |
|  | Neurotrophic Factor Deprivation in Retinal Ganglion Cell Death |
|  | Cytokines Trigger Otitis Media |
|  | MUC5AC Expression by NTHi and S. Pneumoniae |
|  | Immune System Role in Otitis Media |
|  | Alveolar Macrophage Negative Regulation Declined in COPD |
|  | Alveolar Macrophages Dysfunction in COPD |
|  | T-Cell Cytotoxic Activation |
|  | Alveolar Epithelial Cell Dysfunction in COPD |
|  | Mucin Hyperproduction in Goblet and Mucous Cells |
|  | Endothelial Cell Dysfunction in Glomerulonephritis |
|  | Toll-like Receptors in Pyelonephritis |
|  | Endothelial Cell Dysfunction in Pyelonephritis |
|  | CD38/CD19 -> JUN/FOS/NF-kB Signaling in B-cell Proliferation |
|  | B-Cell Receptor -> NF-kB Signaling |
|  | TLR -> ARRB2/NF-kB Signaling |
|  | gamma Globulins Expression Targets |
|  | CD22 -> NF-kB Expression Targets |
|  | CD72 -> STAT Expression Target |
|  | CD8 -> STAT Expression Targets |
|  | CD80 -> STAT Expression Targets |
|  | CD86 -> STAT Expression Targets |
|  | PECAM1 Expression Targets |
|  | NFKB Canonical Signaling Activation in Cancer |
|  | NFKB Non-Canonical Signaling Activation in Cancer |
|  | STAT3 and NFkB Activate Inflammation-Induced Tumorigenesis |
|  | IL23A and IL17A Provoke Cancer-Associated Inflammation |
|  | TGFB Signaling activation by Blocking of Tumor Suppressors |
|  | NFKB Signaling Activation by Blocking of Tumor Suppressors |
|  | TGFB Family in Epithelial to Mesenchymal Transition in Cancer |
|  | IDO1 in Cancer Immune Escape |
|  | IFNLR1-> STAT/NFKB Signaling |
|  | IL1R -> STAT3 Signaling |
|  | IL1R -> NF-kB Signaling |
|  | IL16 -> NF-kB Expression Targets |
|  | CD40 -> Expression Targets in Lymph Node |
|  | NGF -> SMAD3/NF-kB Expression Targets |
|  | NGF -> AP-1/TP53/MYC Expression Targets |
|  | NGF -> FOXO/MYCN/ELK-SRF Expression Targets |
|  | BDNF Expression Targets |
|  | NTF3 Expression Targets |
|  | NTF4 Expression Targets |
|  | NGF -> CREB/CEBPB/MEF2A Expression Targets |
|  | PDCD1 -> NF-kB Expression Targets |
|  | DDR1 -> NF-kB Signaling |
|  | Collagen -> NF-kB Expression Targets |
|  | JNK/MAPK Signaling |
|  | TGF-beta Signaling |
|  | P38 MAPK/MAPK14 Signaling |
|  | NF-kB non-Canonical Signaling |
|  | ERK5/MAPK7 Signaling |
|  | NF-kB Canonical Signaling |
|  | TCR -> NF-kB Expression Targets |
|  | T-Cell Receptor -> AP-1 Signaling |
|  | T-Cell Receptor -> NF-kB Signaling |
|  | CD72 -> AP-1 Expression Targets |
|  | CD40LG -> NF-kB/ELK/SRF -> CREB/NFATC Expression Targets |
|  | TNFSF13 Expression Targets |
|  | TNFSF13B Expression Targets |
|  | EDA Expression Targets |
|  | EctodysplasinR -> NF-kB Signaling |
|  | EctodysplasinR -> AP-1 Signaling |
|  | TNFR -> CREB/ELK-SRF Signaling |
|  | TNFR -> AP-1/ATF/TP53 Signaling |
|  | TNFRSF5/13B -> NFATC1 Signaling |
|  | NGFR -> AP-1/CEBPB/CREB/ELK/SRF/TP53 Signaling |
|  | TNFRSF5/6 -> RB1/E2F Signaling |
|  | NGFR -> NF-kB Signaling |
|  | EctodysplasinR -> LEF1 Signaling |
|  | TNFR -> NF-kB Signaling |
|  | NGFR -> MEF Signaling |
|  | TLR4 -> AP-1 Expression Targets |
|  | TLR5 Expression Targets |
|  | TLR7 Expression Targets |
|  | TLR4 -> AP-1/EGR1/HIF1A Expression Targets |
|  | TLR1 -> 2/6 Expression Targets |
|  | IL1B Expression Targets |
|  | TLR4 -> NF-kB/IRF Expression Targets |
|  | IL1A Expression Targets |
|  | TLR9 Expression Targets |
|  | TLR4/5/7/9 -> NF-kB Signaling |
|  | TLR1/2/6 -> NF-kB Signaling |
|  | TLR -> AP-1 Signaling |
|  | pathway 4 |
|  | Model of PPARG signaling in psoriasis |
|  | Model of PPARG related pathways in psoriasis (short version) |
|  | New Pathway (3) |
|  | sars-cov no binding |
|  | dsRNA induces innate immunity and INFs in alveolar cells |
|  | IFN signaling in alveolar cells |
|  | Innate immunity in Leukocytes |
|  | AGER feed forward loop in innate immunity |
|  | Dendritic Cell Activation in COVID19 |
|  | 1\_Inflammation in keratinocytes in the pathology of acne |
|  | 2\_Acute phase initiation in atopic dermatitis |
|  | 2\_Interleukin-17 and interleukin-22 signaling in psoriasis |
|  | 1\_The onset of atopic dermatitis |
|  | 1\_TNF and IL1B provoke ECM degradation in osteoarthritis |
|  | 1\_Osteoclast-mediated bone resorption in postmenopausal women |
|  | 1\_Osteoclast functional impairment in osteopetrosis |
|  | 2\_Chondrocyte death in osteoarthritis |
|  | 1\_The role of Toll-like receptors in pyelonephritis |
|  | 2\_The role of endothelial cells in pyelonephritis |
|  | 3\_Role of neurotrophic factor deprivation in retinal ganglion cell death |
|  | 1\_Insufficient activation of immune response in the middle ear epithelium cells in otitis media |
|  | 2\_Pathogens stimulate mucins expression in the middle ear |
|  | 3\_Arterial wall inflammation and calcification in atherosclerosis |
|  | 1\_Cigarette smoke causes dysfunction and production of inflammatory mediators in alveolar epithelial cells |
|  | 3\_Role of alveolar macrophages in pulmonary tissue destruction in COPD |
|  | 1\_Th2 cell response in asthma |
|  | 3\_2\_Regulation of airway surface liquid (ASL) thickness |
|  | 3\_1\_Mucus accumulation in asthma |
|  | 2\_2\_Eosinophilia and neutrophilia in asthma: eosinophil apoptosis |
|  | 4\_Mucin hyperproduction in goblet and mucous cells in COPD |
|  | 2\_Paneth cell dysfunction in Crohn's disease |
|  | 1\_Defects in response to pathogens in the gut promotes inflammation in Crohn's disease |
|  | 2\_Intestine epithelial cells drive immunological responses in ulcerative colitis |
|  | 1\_Intestine epithelial cell dysfunction in ulcerative colitis |
|  | 2\_Lipotoxicity in NAFLD |
|  | 1\_CD4+ T cell and epithelial cell response in celiac disease |
|  | 6\_p12I Viral protein promotes T-cell activation in HTLV-1 infection |
|  | 5\_Human papilloma virus E6 and E7 proteins promote epithelial cell proliferation |
|  | 4\_3\_Insulin resistance: FFA-related insulin resistance |
|  | 1\_CFTR expression and degradation in epithelial cells in cystic fibrosis (class I-II mutations) |
|  | 1\_Antigen-presenting cells promote islet dysfunction and immune system activation in T1D |
|  | 3\_Islet beta-cell destruction in T1D |
|  | 2\_Neuron and Schwann cell death |
|  | Figure 4\_Diabetes mellitus Type 1: conventional dendritic cell |
|  | Model of PPARG signaling in psoriais (tested) |
|  | Interleukin-17 and interleukin-22 signaling in psoriasis |

---

|  |  |
| --- | --- |
| Group | other partners for SARS |

---

|  |  |
| --- | --- |
| MedScan ID | 7189 |

---

|  |  |
| --- | --- |
| LocusLink ID | 7189 |
|  | 22034 |
|  | 311245 |
|  | 72304 |
|  | 99098 |

---

|  |  |
| --- | --- |
| Alias | OTTMUSP00000015480 |
|  | C630032O20 |
|  | MGC:3310 |
|  | interleukin-1 signal transducer |
|  | AI851288 |
|  | TNF receptor-associated factor 6, E3 ubiquitin protein ligase |
|  | RING-type E3 ubiquitin transferase TRAF6 |
|  | TNF receptor-associated factor 6 |
|  | LOC100048242 |
|  | RING finger protein 85 |
|  | RNF85 |
|  | TNF receptor-associated factor VI |
|  | E3 ubiquitin-protein ligase TRAF6 |
|  | TNF-alpha receptor-associated factor 6 |
|  | RIKEN cDNA 2310003F17 gene |
|  | LOC100042930 |
|  | C630032O20Rik |
|  | Tumor necrosis factor-alpha receptor associated factor 6 |
|  | TRAF6 |
|  | 2310003F17Rik |
|  | Tumor necrosis factor receptor associated factor 6 |
|  | 2310003F17 |
|  | tumor necrosis factor (TNF) receptor-associated factor 6 |
|  | RP23-313G3.1 |
|  | RIKEN cDNA C630032O20 gene |
|  | tumour necrosis factor receptor associated factor 6 |
|  | tumour necrosis factor (TNF) receptor-associated factor 6 |
|  | Putative interleukin 1 signal transducer |
|  | LOC222344 |
|  | expressed sequence AI851288 |
|  | Tnf receptor-associated factor 6 (predicted) |

---

|  |  |
| --- | --- |
| EC Number | 2.3.2.27 |
|  | 6.3.2.- |

---

|  |  |
| --- | --- |
| GO ID | 0042826 |
|  | 0031435 |
|  | 0047485 |
|  | 0043422 |
|  | 0019901 |
|  | 0031996 |
|  | 0005164 |
|  | 0031624 |
|  | 0061630 |
|  | 0031625 |
|  | 0004842 |
|  | 0008270 |
|  | 0038095 |
|  | 0007249 |
|  | 0007254 |
|  | 0002755 |
|  | 0050852 |
|  | 0042088 |
|  | 0000187 |
|  | 0007250 |
|  | 0032147 |
|  | 0019886 |
|  | 0045453 |
|  | 0048468 |
|  | 0006974 |
|  | 0071345 |
|  | 0071222 |
|  | 0001701 |
|  | 0070498 |
|  | 0031293 |
|  | 0043011 |
|  | 0043066 |
|  | 0000122 |
|  | 0045892 |
|  | 0001843 |
|  | 0070423 |
|  | 0042475 |
|  | 0001503 |
|  | 0030316 |
|  | 0051091 |
|  | 0043123 |
|  | 0043507 |
|  | 0051092 |
|  | 1901224 |
|  | 0002726 |
|  | 0042102 |
|  | 0043065 |
|  | 0045084 |
|  | 0032743 |
|  | 0045410 |
|  | 1904996 |
|  | 0031666 |
|  | 0045672 |
|  | 0031398 |
|  | 0048661 |
|  | 0045944 |
|  | 2000679 |
|  | 0070534 |
|  | 0051865 |
|  | 0016579 |
|  | 0000209 |
|  | 0051023 |
|  | 0070555 |
|  | 0002223 |
|  | 0034162 |
|  | 0002224 |
|  | 0035631 |
|  | 0005938 |
|  | 0005737 |
|  | 0009898 |
|  | 0005829 |
|  | 0010008 |
|  | 0005811 |
|  | 0005634 |
|  | 0048471 |
|  | 0005886 |
|  | 0032991 |
|  | 0046872 |
|  | 0016740 |
|  | 0009887 |
|  | 0046849 |
|  | 0019221 |
|  | 0006955 |
|  | 0002376 |
|  | 0016567 |
|  | 0043122 |
|  | 0042981 |
|  | 0007165 |
|  | 0005622 |
|  | 0045087 |
|  | 0051403 |
|  | 0016874 |
|  | 0002756 |
|  | 0008624 |
|  | 0005515 |
|  | 0035666 |
|  | 0008063 |
|  | 0043234 |
|  | 0048011 |
|  | 0034166 |
|  | 0070740 |
|  | 0070736 |
|  | 0070735 |
|  | 0038123 |
|  | 0070738 |
|  | 0038124 |
|  | 0070737 |
|  | 0070739 |
|  | 0004871 |
|  | 0031965 |
|  | 0005739 |
|  | 0097190 |
|  | 0043773 |
|  | 0043774 |
|  | 0005730 |
|  | 0050870 |
|  | 0006915 |
|  | 0034146 |
|  | 0006916 |
|  | 0035872 |
|  | 0034142 |
|  | 0034134 |
|  | 0034138 |
|  | 0034130 |
|  | 0018169 |
|  | 0006461 |
|  | 0008766 |
|  | 0016564 |
|  | 0005624 |
|  | 0008152 |
|  | 0019941 |
|  | 0004872 |
|  | 0006512 |
|  | 0042591 |

---

|  |  |
| --- | --- |
| KEGG ID | hsa:7189 |
|  | mmu:22034 |
|  | rno:311245 |

---

|  |  |
| --- | --- |
| MedLine Reference | 15890643 |
|  | 16079148 |
|  | 10215628 |
|  | 10349636 |
|  | 10421844 |
|  | 11007897 |
|  | 11042159 |
|  | 11076861 |
|  | 11217851 |
|  | 11250893 |
|  | 11547829 |
|  | 11923871 |
|  | 11967542 |
|  | 11994463 |
|  | 12060722 |
|  | 12140561 |
|  | 12185073 |
|  | 12354380 |
|  | 12459498 |
|  | 12466851 |
|  | 12477932 |
|  | 12566447 |
|  | 12616498 |
|  | 12631284 |
|  | 12637570 |
|  | 12711536 |
|  | 12819136 |
|  | 12902324 |
|  | 12958312 |
|  | 14499111 |
|  | 14500659 |
|  | 14634083 |
|  | 14645259 |
|  | 14661019 |
|  | 14673102 |
|  | 14699584 |
|  | 15102471 |
|  | 15305292 |
|  | 15308666 |
|  | 15322147 |
|  | 15361868 |
|  | 15385464 |
|  | 15548667 |
|  | 15678102 |
|  | 15705807 |
|  | 15864310 |
|  | 15905851 |
|  | 16033648 |
|  | 16141072 |
|  | 16141073 |
|  | 16147974 |
|  | 16237096 |
|  | 16246731 |
|  | 16252010 |
|  | 16263713 |
|  | 16306937 |
|  | 16378096 |
|  | 16393988 |
|  | 16436380 |
|  | 16446357 |
|  | 16630834 |
|  | 16772369 |
|  | 16831874 |
|  | 16897814 |
|  | 16921377 |
|  | 17055451 |
|  | 17092936 |
|  | 8889548 |
|  | 8910514 |
|  | 10025951 |
|  | 10075662 |
|  | 10094049 |
|  | 10383454 |
|  | 10411888 |
|  | 10465784 |
|  | 10514511 |
|  | 10523862 |
|  | 10635328 |
|  | 10747026 |
|  | 10748240 |
|  | 10764746 |
|  | 10880535 |
|  | 10882101 |
|  | 10920205 |
|  | 11035039 |
|  | 11057907 |
|  | 11244088 |
|  | 11278268 |
|  | 11279055 |
|  | 11397809 |
|  | 11406619 |
|  | 11418661 |
|  | 11463333 |
|  | 11607847 |
|  | 11751921 |
|  | 11847341 |
|  | 11882293 |
|  | 11960013 |
|  | 12048196 |
|  | 12193732 |
|  | 12270937 |
|  | 12496252 |
|  | 12609980 |
|  | 12714497 |
|  | 12721283 |
|  | 12761501 |
|  | 12804775 |
|  | 12874243 |
|  | 12917691 |
|  | 12925853 |
|  | 14500648 |
|  | 14530355 |
|  | 14550571 |
|  | 14593105 |
|  | 14633987 |
|  | 14665433 |
|  | 14743216 |
|  | 14754897 |
|  | 14996708 |
|  | 15125833 |
|  | 15247281 |
|  | 15280356 |
|  | 15456887 |
|  | 15492226 |
|  | 15591054 |
|  | 15634933 |
|  | 15665823 |
|  | 15670770 |
|  | 16052631 |
|  | 16153868 |
|  | 16184196 |
|  | 16189514 |
|  | 16196101 |
|  | 16221674 |
|  | 16230348 |
|  | 16251197 |
|  | 16260598 |
|  | 16280329 |
|  | 16313339 |
|  | 16354686 |
|  | 16452479 |
|  | 16484229 |
|  | 16517750 |
|  | 16527194 |
|  | 16920630 |
|  | 16932746 |
|  | 8837778 |
|  | 9374458 |
|  | 9642260 |
|  | 9685412 |
|  | 9915784 |
|  | 9928991 |
|  | 10346818 |
|  | 10908663 |
|  | 11384837 |
|  | 15489334 |
|  | 16125763 |
|  | 16286016 |
|  | 17135271 |
|  | 17346928 |
|  | 17363736 |
|  | 17377523 |
|  | 17572386 |
|  | 9020361 |
|  | 9432981 |
|  | 9774460 |
|  | 9774977 |
|  | 16210631 |
|  | 17224624 |
|  | 17277780 |
|  | 17332487 |
|  | 17360955 |
|  | 17462996 |
|  | 17507094 |

---

|  |  |
| --- | --- |
| Organism | Homo sapiens {Organism urn:agi-taxid:9606} |
|  | Mus musculus {Organism urn:agi-taxid:10090} |
|  | Rattus norvegicus {Organism urn:agi-taxid:10116} |
|  | Homo sapiens |
|  | Mus musculus |
|  | Rattus norvegicus |

---

|  |  |
| --- | --- |
| Mouse chromosome position | 2 |
|  | 2 E2 |

---

|  |  |
| --- | --- |
| OMIM ID | 602355 |

---

|  |  |
| --- | --- |
| Rat chromosome position | 3q31 |

---

|  |  |
| --- | --- |
| Hugo ID | 12036 |
|  | HGNC:12036 |

---

|  |  |
| --- | --- |
| Human chromosome position | 11p12 |

---

|  |  |
| --- | --- |
| Swiss-Prot Accession | Q9Y4K3 |
|  | Q9Y4K3.1 |
|  | P70196 |
|  | P70196.2 |
|  | B5DF45 |
|  | B5DF45.1 |
|  | A6NKI7 |
|  | A8KAB3 |
|  | D3DR16 |
|  | Q8NEH5 |
|  | Q6P9M0 |
|  | Q8BLV2 |
|  | TRAF6\_HUMAN |
|  | B5DF45\_RAT |
|  | Q6P9M0\_MOUSE |

---

|  |  |
| --- | --- |
| PIR ID | S71821 |

---

|  |  |
| --- | --- |
| GenBank ID | NC\_000011 |
|  | NM\_004620 |
|  | NP\_004611 |
|  | NM\_145803 |
|  | NP\_665802 |
|  | XM\_017018220 |
|  | XP\_016873709 |
|  | AC009656 |
|  | AC061999 |
|  | AY228337 |
|  | AAO38054 |
|  | CH471064 |
|  | EAW68119 |
|  | EAW68120 |
|  | EAW68121 |
|  | EAW68122 |
|  | AK128396 |
|  | AK292978 |
|  | BAF85667 |
|  | BC031052 |
|  | AAH31052 |
|  | BI463192 |
|  | BQ187062 |
|  | DA855021 |
|  | U78798 |
|  | AAB38751 |
|  | Q9Y4K3 |
|  | NC\_000068 |
|  | NM\_009424 |
|  | NP\_033450 |
|  | NM\_001303273 |
|  | NP\_001290202 |
|  | AL929571 |
|  | CH466519 |
|  | EDL27656 |
|  | EDL27657 |
|  | AK009110 |
|  | AK033718 |
|  | AK040308 |
|  | AK041172 |
|  | BAC30850 |
|  | AK051130 |
|  | AK083275 |
|  | AK155434 |
|  | BAE33263 |
|  | BC060705 |
|  | AAH60705 |
|  | BY131535 |
|  | D84655 |
|  | BAA12705 |
|  | P70196 |
|  | NC\_005102 |
|  | NM\_001107754 |
|  | NP\_001101224 |
|  | AC\_000071 |
|  | AABR07053106 |
|  | AAHX01023904 |
|  | CH473949 |
|  | EDL79625 |
|  | BC168921 |
|  | AAI68921 |
|  | FQ209645 |
|  | B5DF45 |
|  | CAM20505 |
|  | XP\_006234705 |
|  | XM\_006499156 |
|  | AC\_000024 |
|  | AC\_000143 |
|  | NC\_018922 |
|  | ABM85318 |
|  | AABR06025116 |
|  | AAHY01019032 |
|  | DQ891209 |
|  | ABBA01060847 |
|  | ABBA01060846 |
|  | ABM82135 |
|  | XP\_006499219 |
|  | AMYH02024607 |
|  | DQ894392 |
|  | XM\_006234643 |
|  | NT\_009237 |
|  | NW\_925006 |
|  | NW\_001838022 |
|  | AC\_000054 |
|  | CR590964 |
|  | CR612801 |
|  | NT\_039207 |
|  | NW\_001030694 |
|  | NW\_001084813 |
|  | NW\_047657 |
|  | Q6P9M0 |
|  | XM\_230377 |
|  | XP\_230377 |
|  | XM\_001079281 |
|  | XP\_001079281 |
|  | XM\_001479093 |
|  | XP\_001479143 |
|  | XM\_001479837 |
|  | XP\_001479887 |
|  | BG916928 |
|  | AI414846 |
|  | AV377471 |
|  | NW\_000178 |
|  | 1651195 |
|  | 26334265 |
|  | 30580620 |
|  | 38173732 |
|  | 74206917 |
|  | 81911340 |
|  | AI851288 |
|  | 119588525 |
|  | 119588526 |
|  | 119588528 |
|  | 123995433 |
|  | 1732426 |
|  | 21410269 |
|  | 28372409 |
|  | 30580642 |
|  | 119588527 |
|  | 123980612 |
|  | 123239824 |
|  | 148695709 |
|  | 148695710 |
|  | 149022731 |

---

|  |  |
| --- | --- |
| Swiss-Prot ID | TRAF6\_HUMAN |
|  | TRAF6\_MOUSE |
|  | TRAF6\_RAT |
|  | B5DF45\_RAT |
|  | Q6P9M0\_MOUSE |
|  | D3DR16\_HUMAN |

---

|  |  |
| --- | --- |
| Cell Localization | Cytoplasm |
|  | Cell cortex |
|  | Nucleus |
|  | Lipid droplet |
|  | Cytoplasm, cell cortex |

---

|  |  |
| --- | --- |
| Ensembl ID | ENSG00000175104 |
|  | ENSP00000433623.1 |
|  | ENST00000526995.6 |
|  | ENSP00000337853.5 |
|  | ENST00000348124.5 |
|  | ENSMUSG00000027164 |
|  | ENSMUSP00000004949.7 |
|  | ENSMUST00000004949.7 |
|  | ENSRNOG00000004639 |
|  | ENSRNOP00000006148.4 |
|  | ENSRNOT00000006148.5 |
|  | ENST00000526995 |
|  | ENSMUST00000004949 |
|  | ENSP00000337853 |
|  | ENSMUSP00000004949 |
|  | ENST00000526995.5 |
|  | ENSP00000433623 |
|  | ENSRNOP00000006148 |
|  | ENST00000348124 |
|  | ENSRNOT00000006148 |

---

|  |  |
| --- | --- |
| MGI ID | MGI:108072 |
|  | 108072 |
|  | 2139081 |
|  | 1919554 |

---

|  |  |
| --- | --- |
| RGD ID | 1306853 |

---

|  |  |
| --- | --- |
| Unigene ID | Mm.292729 |
|  | Rn.24348 |
|  | Hs.591983 |
|  | Rn.220435 |
|  | Hs.444172 |
|  | Mm.460024 |
|  | Rn.106292 |
|  | Mm.439908 |

---

|  |  |
| --- | --- |
| Homologene ID | 3395 |

---

|  |  |
| --- | --- |
| IPI ID | IPI00136488 |
|  | IPI00222919 |
|  | IPI00743663 |
|  | IPI00363697 |
|  | IPI00420463 |

---

|  |  |
| --- | --- |
| Homo sapiens Chromosome position | 11p12 |

---

|  |  |
| --- | --- |
| Ensembl Transcript ID | ENST00000348124 |
|  | ENST00000313105 |
|  | ENST00000526995 |

---

|  |  |
| --- | --- |
| FunctionalClass | protein binding |
|  | signal transducer |
|  | metal ion binding |
|  | receptor |
|  | zinc ion binding |
|  | ubiquitin-protein ligase |

---

|  |  |
| --- | --- |
| Mus musculus Chromosome position | 2 E2 |
|  | 2 |

---

|  |  |
| --- | --- |
| Rattus norvegicus Chromosome position | 3q31 |

---

|  |  |
| --- | --- |
| Ensembl Gene ID | ENSG00000175104 |

---
